# Association of sociodemographic and lifestyle factors with dietary patterns among men and women living in Mexico City: A cross-sectional study

**DOI:** 10.3389/fpubh.2022.859132

**Published:** 2022-08-11

**Authors:** Cecilia Isabel Oviedo-Solís, César Hernández-Alcaraz, Néstor Alonso Sánchez-Ortíz, Nancy López-Olmedo, Alejandra Jáuregui, Simón Barquera

**Affiliations:** ^1^Center of Research in Nutrition and Health, National Institute of Public Health, Cuernavaca, Mexico; ^2^Center for Population Health Research, National Institute of Public Health, Cuernavaca, Mexico

**Keywords:** dietary patterns, sociodemographic factors, lifestyle factors, Mexico City, sex differences, representative survey

## Abstract

**Background:**

Diet is one of the leading risk factors for non-communicable diseases and is related to sociodemographic and lifestyle factors, including sex. These associations vary across populations. We aimed to investigate which factors are associated with dietary patterns among adults living in Mexico City by sex.

**Methods:**

We used data from the Mexico City Diabetes Representative Study, a cross-sectional, multistage, stratified, and cluster-sampled survey in Mexico City (*n* = 1,142; 413 men and 729 women). Dietary information was collected using a semi-quantitative food frequency questionnaire. Foods and beverages were categorized into 23 food groups to identify dietary patterns by cluster analysis. Sociodemographic and lifestyle variables included were self-reported through standardized questionnaires. We assessed the association of sociodemographic and lifestyle factors with dietary patterns through a multinomial logistic model stratified by sex.

**Results:**

We identified three dietary patterns: basic, prudent, and fast food. Among men and women, higher school attainment was associated with a lower relative probability of having a basic rather than prudent dietary pattern (women: RRR = 0.8, 95% CI: 0.8, 0.9; men: RRR = 0.8, 95% CI: 0.7, 0.9). Divorced or separated men (RRR = 3.8, 95% CI: 1.3, 11.2) and those living with a partner (RRR = 2.6, 95% CI: 1.1, 6.1) had a higher relative probability of consuming a fast food dietary pattern than the prudent one, compared to single men. Men living with a partner (RRR = 3.0, 95% CI: 1.1, 8.6) or working long shifts (RRR = 3.8, 95% CI: 1.3, 11.1) had a higher probability of consuming a basic pattern rather than a prudent one compared to peers. Among women, those with high SES had a lower probability of consuming the “basic” pattern rather than the “prudent” pattern compared to those with low SES. No lifestyle factors were associated to dietary patterns.

**Conclusions:**

Men living in Mexico City with lower education, age, non-single, and working long hours (i.e., more than the established by the law), and women with lower age, education, and socioeconomic level are prone to adhere to unhealthy diets. These associations are likely to be driven by gender roles.

## Introduction

Unhealthy diets are one of the major lifestyle risk factors for non-communicable diseases (NCDs), which account for more than 75% of the deaths worldwide (1, 2). In Mexico, unhealthy diets contribute to 11.3% of total deaths (3). Other modifiable risk factors, such as physical inactivity, sedentary behaviors (i.e., sitting time, screen time), tobacco use, and excessive alcohol use also contribute to the burden of NCDs (2). These risk factors are influenced by lifestyle and gender roles, and may be correlated with each other (4). The characterization of dietary patterns through sociodemographic and lifestyle factors may contribute to identifying target populations for nutrition-related interventions, programs, and policies (5, 6).

Differences by sex in dietary patterns have been described previously in the Mexican population (7–10). It has been observed that the dietary patterns of women include healthier foods such as vegetables and fruits and more sweets and cakes. In contrast, men eat more red and processed meats, sugar-sweetened and alcoholic beverages (7–9). These patterns are potentially associated with other lifestyle and sociodemographic factors. To our knowledge, the association of lifestyle and sociodemographic factors with dietary patterns by sex has not been analyzed in the Mexican population.

The aim of this study was to evaluate the association of sociodemographic and lifestyle factors with dietary patterns by sex of adults living in Mexico City in 2015, using the Mexico City Diabetes Representative Study.

## Methods

### Design and study population

We conducted a secondary study using data come the Mexico City Diabetes Representative Study, a cross-sectional, multistage, stratified, and cluster-sampled survey, conducted between May and June 2015 in Mexico City. Census tracts, defined by National Institute of Geographics and Informatics, were the primary sampling units. Details of sampling procedures are described elsewhere (11). Briefly, two adults aged 20–69 years old were systematically selected in each house. Trained personnel collected information through face-to-face interviews. The response rate for the original study was 69%. Information on 1,334 adults was collected. Adults with invalid or incomplete information on lifestyle and sociodemographic variables were excluded from this analysis, as well as individuals with extreme total energy intake (with energy intake/basal metabolic rate <0.05 or energy intake/estimated energy intake ratio >3 S.D.), as previously described (12). Thus, the present study considered 1,142 individuals, 36.2% men and 63.8% women.

### Sociodemographic and lifestyle factors

Sociodemographic variables were measured using validated questions from the National Health and Nutrition Survey in Mexico (13), including age (in years), sex (male; female), years of school attainment (in years), socioeconomic status (SES) (low; middle; high), marital status (single; living with a partner; divorced or separated; and widowed), and employment status (unemployed; working 48 h or less per week; working more than 48 h per week) (14) (see **Table 2**). SES was constructed using principal components analysis (PCA). Household characteristics (flooring material, ceiling, walls, water source, sewerage, number of persons residing in the household, and domestic appliances) were entered into the PCA. The main factor was extracted and divided into tertiles (as a proxy for low, middle, and high SES). The detailed methodology is described elsewhere (15).

Four categories were considered for marital status: single, living with a partner, divorced or separated, and widowed. Working hours per week were categorized considering legal working hours per week in Mexico as the cut-off point (48 h per week) (14).

Lifestyle variables included smoking status, sleep duration, alcohol use, sitting time, and physical activity volume. Smoking status was categorized as never, former, and current. Healthy sleep duration considers people reporting sleeping 7 h or more, while unhealthy consider people reporting <7 h (16).

Alcohol use was classified into three categories: never, low (five drinks or less per week), and high (more than five drinks per week).

Physical activity volume was measured using the short version of the International Physical Activity Questionnaire (IPAQ). The IPAQ consists of 7 questions that measure moderate to vigorous physical activity, walking, and sitting time. Data were processed following the IPAQ Analysis guidelines (17), and metabolic equivalent (MET)-minutes per week of total physical activity were calculated. Participants were classified as with low (<600 MET-minutes/week or without activity reported), moderate (600–1,500 MET-minutes/week), and high levels (≥1,500 MET-minutes/week) of physical activity.

Sitting time was measured as an indicator of sedentary behavior. Hours and minutes of sitting time in 1 day of the last 7 days were asked. Daily sitting hours were estimated.

### Dietary patterns

Diet information was collected with a validated semi-quantitative food frequency questionnaire (SFFQ) (18). The SFFQ assessed the consumption of 140 food and beverage items during the last 7 days. The frequency of food consumption was reported using pre-established categories ranging from never to six times a day. The SFFQ standard portion sizes specified by interviewers were based on the average weight value assigned to each food item. We first converted data from the SFFQ to times per day, and then the portion size per day was calculated. Mean food item servings per day and daily energy intake (kcal/day) were calculated using the food composition tables compiled by the National Institute of Public Health (12).

To identify dietary patterns, we categorized foods and beverages from the SFFQ into 23 food groups (Table 1). Food groups were defined by the similarity of nutrient profiles (e.g., the proportion of lipids, proteins, carbohydrates, or dietary fiber) or the amount of added sugar (e.g., sweetened beverages). Tortillas were considered as a sole component because of their culinary use in Mexico. All food items were placed in a specific food group. We calculated the contribution of each food group to the total energy intake as percentages and standardized using z-scores. The k-means method was used in a cluster analysis to derive dietary patterns based on the food group's contribution (19, 20). Two to five solutions were tested. The solution that best discriminated across groups while maintaining enough cases in each group was selected. We also considered the Calinski-Harabasz criterion (21) to determine the optimal number of clusters. We used the percentage energy contribution that significantly differed from other food groups between clusters to define dietary patterns. Patterns were named based on the major food groups and nutrients that characterized each group relative to others and considered a minimum of 0.30 in z-score.

**Table 1:** Percent contribution to total energy intake from food groups by dietary pattern.

**Dietary pattern**	**Total**	**Prudent**	**Fast food**	**Basic**
**Food group**			**%**
Dairy (Not sweetened)	8.17	13.07	6.94	5.94
Cereals ready to consumption	0.81	1.92	0.5	0.34
Chicken and egg	5.92	6.68	4.38	7.01
Fast food	3.37	2.66	5.37	1.73
Fats	0.91	0.61	1.38	0.62
Fish and seafood	0.66	1.5	0.36	0.36
Flavored and sweetened dairy products	1.68	1.81	1.41	0.87
Fruit and vegetables juices	2.62	4.02	2.03	2.22
Fruits	6.18	10.02	4.7	4.96
Legumes	1.32	1.81	0.95	2.36
Corn tortillas	17.14	10.42	11.37	28.24
Corn -based foods	6.73	4.18	9.99	5.1
Oilseeds	2.02	3.56	1.79	1.15
Red and processed meat	5.36	4.46	4.9	6.52
Rice and potatoes	3.55	3.64	2.86	4.24
Salty snacks	1.96	1.74	2.66	1.36
Soups, broths and pasta	3.95	4.32	3.06	4.63
Sweetened beverages	6.46	3.28	9.19	5.84
Sweetened cereals	8.68	6.45	12.84	5.83
Sweets and desserts	5.09	4.17	6.98	3.74
Vegetables	2.1	3.36	1.43	1.89
Whole-grain bread	1.19	3.17	0.64	0.33
White bread and flour tortilla	4.15	3.15	4.29	4.72

### Statistical analysis

The resulting dietary patterns were described according to the mean energy contribution (%). We first descriptively examined the sociodemographic and lifestyle variables in the total sample and by sex. We then assessed the adjusted relative risk ratio (RRR) of belonging to a dietary pattern through a multinomial logistic model stratified by sex. We considered the dietary patterns as the outcome variable using the healthier pattern as the reference category, and sociodemographic (age, sex, years of school attainment, socioeconomic status, marital status, and employment status), and lifestyle factors (smoking status, sleep duration, alcohol use, sitting time, and physical activity level) as independent variables. The established significance level was at alpha 0.05. All the analyses were performed using expansion weights and adjusted by survey design with the SVY module of Stata 14^®^.

## Results

Three main dietary patterns were identified. The first pattern had the highest contribution from foods commonly considered healthy (i.e., unsweetened dairy, fruits, oilseeds, vegetables, and whole-grain); therefore, we named this pattern “prudent.” Sweetened cereals, corn-based foods, sweetened beverages, sweets and desserts, and fast food contributed heavily to the second pattern; we named this pattern “fast food.” The third pattern was characterized by high consumption of corn-tortillas and legumes, which are basic elements of the Mexican diet; this pattern was named “basic” (Table 1).

In the total sample, the most consumed pattern was the “fast food” (41.5%), and the less consumed was the “prudent” pattern (24.9%); we observed similar distributions by sex. Participants were, on average, 40.1 years old and had 12.5 years of school attainment. More than half were from the low and middle socioeconomic status (58.0%) and lived with a partner (55.9%). In the total sample, 38.3% were unemployed, with a higher unemployment rate among females (51.3%) compared to males (22.5%) (Table 2).

**Table 2:** Sociodemographic and lifestyle characteristics of study participants by sex in Mexico City, 2015.

	**Total sample**	**Men**	**Women**
	***n =*** **1,142**	***n =*** **413**	***n =*** **729**
**Total**		45.1 (41.5, 48.8)	54.9 (51.2, 58.5)
**Dietary pattern**, ***n*****, % (95%CI)**			
Prudent	298, 24.9 (21, 29.3)	95, 21.9 (17, 27.6)	203, 27.4 (22.5, 32.9)
Fast food	437, 41.5 (37.1, 46.1)	161, 43.2 (37.5, 49.2)	276, 40.1 (35.1, 45.4)
Basic	407, 33.6 (29, 38.5)	157, 34.9 (29, 41.3)	250, 32.5 (27.4, 37.9)
**Sociodemographic variables**			
Age (years), mean (95%CI)	40.1 (39.1, 41.1)	39.3 (37.7, 40.8)	40.7 (39.6, 41.9)
School attainment (years), mean (95%CI)	12.5 (12.1, 12.9)	12.8 (12.3, 13.3)	12.2 (11.8, 12.7)
**Socioeconomic status, % (95%CI)**			
Low	20.8 (17.3, 24.7)	17.3 (13, 22.5)	23.6 (19.8, 28)
Middle	37.2 (32.8, 41.9)	35.9 (29.8, 42.5)	38.2 (33, 43.8)
High	42.1 (36.6, 47.8)	46.8 (39.6, 54.2)	38.1 (32.5, 44.1)
**Marital status, %(95%CI)**			
Single	30.9 (27.4, 34.6)	36.8 (30.7, 43.3)	26.1 (22.1, 30.4)
Living with a partner	55.9 (51.4, 60.3)	55.1 (48.8, 61.2)	56.6 (51.1, 62)
Divorced/Separated	9.6 (7.8, 11.8)	7 (4.7, 10.5)	11.7 (9.1, 14.9)
Widowed	3.6 (2.7, 4.8)	1.1 (0.5, 2.3)	5.7 (4.2, 7.5)
**Employment status^a^, %(95%CI)**			
Unemployed	38.3 (34.5, 42.3)	22.5 (17.3, 28.8)	51.3 (46.3, 56.3)
Working 48 h or less per week	38.1 (33.8, 42.7)	39.1 (32.3, 46.4)	37.3 (32.4, 42.5)
Working more than 48 h per week.	23.5 (20.3, 27.1)	38.4 (32.6, 44.4)	11.3 (8.7, 14.7)
**Lifestyle variables**			
**Smoking status^b^, % (95%CI)**			
Never	43.1 (40, 46.4)	29.2 (24.3, 34.6)	54.6 (49.4, 59.7)
Former	25.7 (22.5, 29.1)	27.2 (22.2, 33)	24.4 (20.6, 28.7)
Current	31.2 (27.7, 34.9)	43.6 (37.9, 49.5)	21 (17.1, 25.5)
**Sleep duration^c^, % (95%CI)**			
Healthy (7 or more hours)	69.8 (64.8, 74.4)	67.3 (59.7, 74.1)	71.8 (66.8, 76.3)
Unhealthy (<7 h)	30.2 (25.7, 35.2)	32.7 (25.9, 40.3)	28.2 (23.7, 33.2)
**Alcohol use, % (95%CI)**			
Never	18.9 (16, 22.2)	10 (7.1, 14)	26.2 (22.2, 30.6)
5 drinks/week or less	75.7 (71.7, 79.3)	79.4 (73.1, 84.6)	72.7 (68.1, 76.8)
More than 5 drinks/week	5.4 (3.9, 7.5)	10.6 (7.4, 14.9)	1.2 (0.6, 2.3)
Sitting time (h/day), mean (95%CI)	3.9 (3.7, 4.1)	4.2 (3.9, 4.5)	3.6 (3.4, 3.9)
**Physical activity^d^, % (95%CI)**			
Low	8.9 (7.1, 10.9)	11.4 (8.7, 14.9)	6.7 (4.8, 9.4)
Moderate	3.7 (2.5, 5.3)	2.6 (1.3, 5.1)	4.6 (2.8, 7.4)
High	87.5 (84.9, 89.7)	86 (82.4, 89)	88.7 (84.5, 91.8)

a Employment status was defined based on the answers to two questions. First, participants were asked if they had worked at least for an hour in the last week. Those who answered “no” were considered as the reference category. Those who answered “yes” were asked, “How many hours per week do you spend in your main job?.” Working hours per week were categorized considering legal working hours per week in Mexico as the cut-off point (48 h per week).

b Smoking status was assessed considering the questions (1) “Have you smoked 100 cigarettes or more in your life?” and (2) “Are you currently smoking?.” Current smokers were those who answered “yes” to both questions; ever smokers answered “yes” to question (1) and “no” to question (2); and never smokers answered “no” to both questions.

c Sleep duration was defined using the question “On average, how many hours do you sleep per day?” with five possible answers: 5 or less, 6, 7, 8, and 9 or more hours. Sleep duration was categorized as sleeping <7 h per day (unhealthy) and ≥7 h per day (healthy).

d Physical activity defined by the short version of the International Physical Activity Questionnaire categories.

Almost a third were current smokers (31.2%); the prevalence was higher in men (43.6%) than women (21.0%). In total, 30.2% of participants reported sleeping <7 h per day, and three in every four reported consuming five or fewer alcoholic drinks per week. On average, participants reported 3.9 h per day of sitting time and only 8.9% reported low physical activity levels (Table 2).

Table 3 shows the adjusted and sex-stratified associations of sociodemographic and lifestyle factors with dietary patterns. In the total sample, a lower probability of consuming the “basic” and the “fast food” patterns than the “prudent” pattern was observed as age and school attainment increased. These associations were consistent in man and women. In the total sample, living with a partner or being divorced/separated (vs. being single) were associated with a higher probability of consuming a “fast food” pattern, while living with a partner (vs. being single) and working more than 48 h per week (vs. being unemployment) were associated with a higher probability of consuming a “basic” pattern rather than a “prudent.” These associations were consistent with those observed in men only. Women with high SES (vs. low SES) had a lower probability of consuming the “basic” pattern rather than the “prudent” pattern.

**Table 3:** Adjusted and sex-stratified association between sociodemographic and lifestyle factors with dietary patterns (Mexico City, 2015).

	**Total sample *n =* 1,142**				**Men, *n =* 413**				**Women, *n =* 729**			
**Sociodemographic variables**	**Fast food^b^(*n =* 437)**		**Basic^b^(*n =* 407)**		**Fast food^b^(*n =* 161)**		**Basic^b^(*n =* 157)**		**Fast food^b^(*n =* 276)**		**Basic^b^(*n =* 250)**	
	**RRR^a^ (95%CI)**	***p***		***p***	**RRR^a^ (95%CI)**	***p***	**RRR^a^ (95%CI)**	***p***	**RRR^a^ (95%CI)**	***p***	**RRR^a^ (95%CI)**	***p***
Women vs. Men	0.8 (0.5, 1.3)	0.333	0.7 (0.4, 1.3)	0.309								
Age (years)	0.9 (0.9, 1.0)	<0.001	1.0 (0.9, 1.0)	<0.001	0.9 (0.9, 0.9)	<0.001	1.0 (0.9, 1.0)	0.021	0.9 (0.9, 1.0)	<0.001	0.9 (0.9, 1.0)	<0.001
School attainment (years)	0.9 (0.9, 1.0)	0.028	0.8 (0.8, 0.9)	<0.001	0.9 (0.8, 1.0)	0.285	0.8 (0.7, 0.9)	<0.001	0.9 (0.9, 1.0)	0.044	0.8 (0.8, 0.9)	<0.001
**Socioeconomic status**													
Low	1.0		1.0		1.0		1.0		1.0		1.0	
Middle	1.1 (0.6, 1.8)	0.811	1.1 (0.7, 1.8)	0.635	1.0 (0.4, 2.4)	0.931	1.2 (0.5, 2.9)	0.632	1.0 (0.6, 1.7)	0.892	1.2 (0.7, 2.2)	0.451
High	1.0 (0.5, 2.0)	0.988	0.6 (0.3, 1.2)	0.163	1.2 (0.4, 3.4)	0.684	1.2 (0.4, 3.5)	0.706	0.9 (0.4, 1.9)	0.794	0.5 (0.3, 1.0)	0.042
**Marital status**													
Single	1.0		1.0		1.0		1.0		1.0		1.0	
Living with a partner	1.7 (1.1, 2.7)	0.031	2.7 (1.5, 4.7)	0.001	2.6 (1.1, 6.1)	0.025	3.0 (1.1, 8.6)	0.039	1.4 (0.8, 2.7)	0.259	1.9 (1.0, 3.6)	0.064
Divorced/Separated	2.2 (1.0, 4.9)	0.045	1.9 (0.9, 4.1)	0.086	3.8 (1.3, 11.4)	0.018	2.9 (0.8, 10.4)	0.111	1.8 (0.6, 5.0)	0.259	1.4 (0.5, 3.6)	0.492
Widowed	3.1 (1.1, 8.7)	0.029	1.9 (0.7, 5.5)	0.214	2.9 (0.2, 38.7)	0.408	0.9 (0.1, 14.5)	0.926	2.5 (0.7, 8.4)	0.140	1.8 (0.5, 6.2)	0.322
**Employment status^c^**													
Unemployed	1.0		1.0		1.0		1.0		1.0		1.0	
Working 48 h or less per week	1.1 (0.7, 1.8)	0.623	1.0 (0.6, 1.5)	0.867	1.1 (0.5, 2.4)	0.865	2.0 (0.8, 4.9)	0.123	1.1 (0.6, 1.9)	0.694	0.7 (0.4, 1.1)	0.119
Working more than 48 h per week.	1.1 (0.6, 2.0)	0.633	1.8 (1.0, 2.9)	0.034	1.1 (0.4, 2.6)	0.873	3.8 (1.3, 11.1)	0.015	1.0 (0.5, 2.2)	0.958	1.0 (0.5, 2.0)	0.892
**Lifestyle variables**												
Smoking status^d^												
Never	1.0		1.0		1.0		1.0		1.0		1.0	
Former	0.9 (0.5, 1.6)	0.715	1.1 (0.6, 2.0)	0.745	1.2 (0.5, 3.1)	0.691	1.0 (0.4, 2.4)	0.924	0.8 (0.4, 1.4)	0.452	1.3 (0.6, 2.5)	0.499
Current	1.4 (0.7, 2.6)	0.294	1.2 (0.7, 2.3)	0.469	2.1 (0.7, 6.2)	0.176	1.9 (0.7, 5.6)	0.212	1.0 (0.5, 1.9)	0.968	0.7 (0.4, 1.5)	0.395
**Sleep duration^e^**													
Unhealthy	1.0		1.0		1.0		1.0		1.0		1.0	
Healthy	0.68 (0.4, 1.0)	0.070	1.0 (0.7, 1.5)	0.993	0.9 (0.5, 1.9)	0.805	1.1 (0.5, 2.4)	0.830	0.6 (0.3, 1.0)	0.052	0.93 (0.5, 1.6)	0.820
**Alcohol use**												
Never	1.0		1.0		1.0		1.0		1.0		1.0	
5 drinks/week or less	1.2 (0.7, 2.0)	0.565	1.0 (0.6, 1.8)	0.945	2.1 (0.6, 7.6)	0.232	1.8 (0.5, 7.2)	0.397	1.0 (0.6, 1.8)	0.950	0.8 (0.4, 1.6)	0.559
More than 5 drinks/week	1.4 (0.5, 4.3)	0.605	1.2 (0.4, 3.8)	0.761	2.2 (0.4, 11.5)	0.338	2.7 (0.6, 10.9)	0.169	0.7 (0.1, 4.4)	0.700	0.1 (0.01, 2.2)	0.145
Sitting time (h/day)	1.0 (0.9, 1.0)	0.385	1.0 (0.9, 1.0)	0.251	0.9 (0.8, 1.0)	0.191	0.9 (0.8, 1.0)	0.162	1.0 (0.9, 1.1)	0.989	1.0 (0.9, 1.1)	0.818
**Physical activity^f^**												
Low	1.0		1.0		1.0		1.0		1.0		1.0	
Moderate	0.7 (0.2, 2.4)	0.580	0.8 (0.2, 2.6)	0.656	1.4 (0.1, 21.4)	0.828	1.2 (0.1, 26.0)	0.892	0.7 (0.3, 2.2)	0.590	0.6 (0.2, 2.1)	0.444
High	1.2 (0.5, 2.6)	0.664	0.9 (0.4, 2.1)	0.897	1.1 (0.3, 4.5)	0.907	1.0 (0.3, 3.5)	0.969	1.5 (0.7, 3.0)	0.249	1.0 (0.4, 2.3)	0.962

Sample sizes for prudent pattern were 298, 95, and 203 in the total sample, men and women, respectively.

a Models were adjusted by all sociodemographic and lifestyle variables. Reference category in adjusted and sex-stratified models was the prudent pattern.

b Dietary patterns defined by cluster analysis. Prudent pattern was used as reference.

c Employment status was defined based on the answers to two questions. First, participants were asked if they had worked at least for an hour in the last week. Those who answered “no” were considered as the reference category. Those who answered “yes” were asked, “How many hours per week do you spend in your main job?.” Working hours per week were categorized considering legal working hours per week in Mexico as the cut-off point (48 h per week).

d Smoking status was assessed considering the questions (1) “Have you smoked 100 cigarettes or more in your life?” and (2) “Are you currently smoking?.” Current smokers were those who answered “yes” to both questions; ever smokers answered “yes” to question (1) and “no” to question (2); and never smokers answered “no” to both questions.

e Sleep duration was defined using the question “On average, how many hours do you sleep per day?” with five possible answers: 5 or less, 6, 7, 8, and 9 or more hours. Sleep duration was categorized as sleeping <7 h per day (unhealthy) and ≥7 h per day (healthy).

f Physical activity defined by the short version of the International Physical Activity Questionnaire categories.

## Discussion

We identified three major dietary patterns in the surveyed population in Mexico City: prudent, fast food, and basic. These dietary patterns were similar to those found in other studies in Mexico (7, 18). While several demographic factors were associated with dietary patterns among men and women, no lifestyle factor was related to dietary patterns in this sample, rejecting our hypothesis that dietary patterns are associated with some lifestyle factors such as smoking status, sleep duration, alcohol use, sitting time, and physical activity.

Studies exploring associations between dietary patterns and lifestyle variables are sparse. Previous reports showed associations between unhealthy lifestyles, such as current smokers (22), sedentary behavior (23), or poor sleep (24), with unhealthy diets. Furthermore, a nationally representative study in Mexico showed that the “refined foods and sweets” pattern had the highest alcohol contribution (7). On the other hand, previous studies have consistently reported associations between physical activity and dietary patterns with high intakes of fruits and vegetables, dairy, nuts, and whole grains, or components of a healthy diet (5, 25–31). However, the results of this study do not show an association between lifestyle variables and dietary patterns. Differences of previous reports can be to lack variability in lifestyle variables in Mexico City population.

The results showed associations between demographic factors and dietary patterns in men and women. School attainment and age were associated with dietary patterns among both men and women. These results are consistent with studies reporting positive associations between higher education (32, 33) and age (5, 25–31) with healthier dietary patterns. Higher education has been associated with increased nutrition knowledge and higher compliance with dietary recommendations (32, 33). The observed association between older age and healthier dietary patterns might be explained by the increased concern about health or the prevention or treatment of non-communicable disease.

Associations between dietary patterns and marital status were observed only in men. Although explaining the findings stratified by sex may be challenging because our results are based on a sex variable, these associations may respond to gender role constructs. Mexican men have been traditionally considered as “providers.” On the other hand, women tend to be responsible for family care and wellbeing (34, 35), with an important role as wives and mothers (28, 29). It has been observed that married couples engage in the same diet (36). We observed that men living with a couple adhere to the basic and fast food pattern, while these associations were not observed in women. We found similar but stronger associations for divorced or separated men.

Similarly, working for more than 48 h was associated with a higher probability of having a basic pattern in men, but not women. The basic pattern identified in our study has characteristics of a “*comida corrida*” in Mexico. The “*comida corrida*” is a cheap home-cooked lunch (<3 USD) available in “*fondas*” or small restaurants that cater to Mexican working-class adults. Due to the high commuting times in Mexico City, most people have to eat out of the home, making this food option a very affordable and accessible one, especially for those that work long periods. However, working hours may not represent shifts or type of work and only paid jobs were considered for the analysis. This may exclude women working at home or self-employed adults.

We observed that a high SES was associated with a healthier dietary pattern among women. In men, we did not find an association between SES and dietary pattern. These results are consistent with those reported in high-income countries (37, 38). The possible explanation is that women are more interested in a better diet and, those who are at high socioeconomic levels, have the resources to choose healthier diets. In a recent study in Mexican women at a low socio-economic level, it has been shown that having limited access and economic resources may shape unhealthy habits (39).

Our study provides an overview of the dietary behavior of Mexico City residents in 2015, although this panorama may have changed in several ways in recent years. There is evidence that the COVID-19 lockdown in 2020 (40) has represented an important shift in consumption patterns in Mexico. Factors such as reductions in mobility and income, as well as increases in food prices and alterations in the food supply chain, have led to changes in food consumption (41, 42). However, the long-term effects are still under analysis. We know that the first wave of COVID-19 was associated with increased food insecurity, particularly among the most vulnerable households (43). In our study, unhealthy dietary patterns (Basic and Fast Food) were associated with more precarious social circumstances (i.e., lower education, older age, being women). Therefore, it is possible that the prevalence of consumption of unhealthy dietary patterns increased during and after the COVID-19 pandemic lockdown.

There are some limitations to the current study. First, even though dietary patterns offer a broader vision of the way populations eat, they are specific to the population studied, limiting the comparability of findings. Second, temporal associations cannot be inferred due to the inherent limitation of the cross-sectional design of this survey. Third, the gender roles discussed focus on the traditional male-female couple model. Future studies will be necessary to better understand the potential role of gender in same-sex couples in dietary patterns. Finally, all the information was self-reported, so we do not rule out the possibility of measurement error (44–46). Specifically, it has been reported that the IPAQ short form overestimates compared with the gold standard (accelerometer data). However, there is a high agreement of categories between questionnaire and objective measures and between total energy expenditure and categories within the vigorous category (47, 48). However, a non-differential error can be expected from our study since the participants did not know the study hypothesis. Strengths include the representative sample of adults living in Mexico City and the use of validated questionnaires, including the SFFQ. Additionally, potential confounders were considered in the analysis. Dietary patterns identified in this study were similar to those found in other studies in Mexico (7, 18). Despite the limitations, the results of this study provide an overview of the association between sociodemographic and lifestyle factors with dietary patterns in men and women living in Mexico City.

## Conclusions

Men living in Mexico City with lower education, age, non-single, and working long hours (i.e., more than the established by the law), and women with lower age, education, and socioeconomic level are prone to adhere to unhealthy diets. These associations are likely to be driven by gender roles due to the differences observed in men and women. The design of nutrition programs should consider gender perspectives to accomplish positive results in men and women.

## Data availability statement

The data analyzed in this study is subject to the following licenses/restrictions: Data could be accessed under permission of the principal investigator of the study. Requests to access these datasets should be directed to AJ, alejandra.jauregui@insp.mx.

## Ethics statement

The studies involving human participants were reviewed and approved by the Ethics Committee of the National Institute of Public Health (Instituto Nacional de Salud Pública, INSP, ID CI1295). The patients/participants provided their written informed consent to participate in this study.

## Author contributions

CO-S, CH-A, and NS-O developed research questions, analyzed, interpreted the data, and wrote the first draft of the manuscript. NL-O and AJ contributed data collection and supported to analyze the data. SB support to interpreted the data and assisted in the writing of the manuscript. All authors read and approved the final manuscript.

## Funding

This study was funded by the Robert Wood Johnson Foundation (RWJF) (Award number: 74155) and Bloomberg Philanthropies (Award number: 43003). The Mexican Ministry of Health provided additional funding through a collaboration agreement with Novo-Nordisk. The National Council for Science and Technology supported CIOS, CHA, and NASO's time writing the paper. The study sponsors had no role in the study design; collection, analysis, and interpretation of data; writing the report; and the decision to submit the report for publication.

## Conflict of interest

The authors declare that the research was conducted in the absence of any commercial or financial relationships that could be construed as a potential conflict of interest.

## Publisher's note

All claims expressed in this article are solely those of the authors and do not necessarily represent those of their affiliated organizations, or those of the publisher, the editors and the reviewers. Any product that may be evaluated in this article, or claim that may be made by its manufacturer, is not guaranteed or endorsed by the publisher.
